# Chemistry in Extreme Environments: The Mystery of Molecular Complexity in Space

**DOI:** 10.1021/acscentsci.5c02122

**Published:** 2026-01-30

**Authors:** Cristina Puzzarini, Silvia Alessandrini

**Affiliations:** Dipartimento di Chimica “Giacomo Ciamician”, Alma Mater Studiorum - University of Bologna, Via P. Gobetti 85, I-40129 Bologna, Italy

## Abstract

Molecular complexity in the interstellar medium (ISM) poses one of the most intriguing challenges in astrochemistry: how can chemical reactions operate efficiently under the extreme physical conditions of space? In this Outlook, we summarize recent advances in understanding the molecular synthesis in the ISM, emphasizing the interplay between gas-phase and grain-surface chemistry. Laboratory studies, ranging from gas-phase kinetics at low temperature to the irradiation of interstellar ice analogues, demonstrate that both energetic and nonenergetic processes contribute to the formation of complex organic and prebiotic molecules. We discuss how accurate exploration of reactive potential energy surfaces by means of quantum-chemical methodologies combined with kinetic simulations provide an atomistic interpretation of the interstellar processes. Despite the advances of the past decade, interstellar chemistry remains in its infancy: reaction networks are incomplete, and quantitative predictions remain limited.

## Graphical abstract

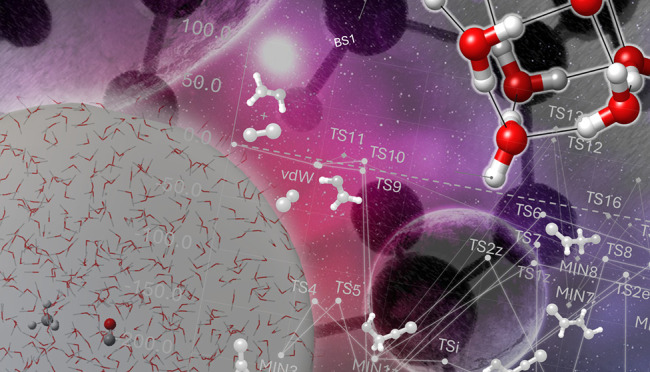

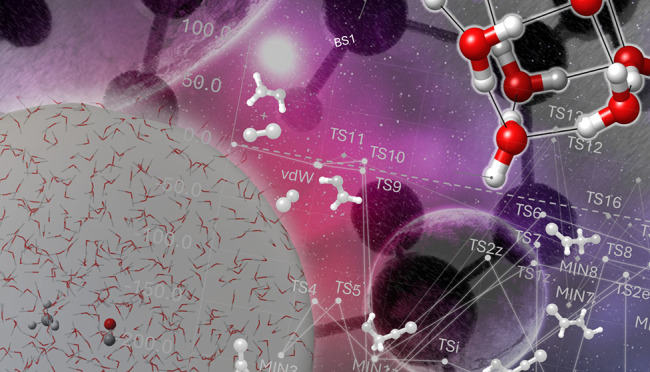

## Introduction

Molecular complexity has been detected across a variety of cosmic environments,^1,2^ from comets and planetary atmospheres to protoplanetary disks.^3–5^ However, the interstellar medium (ISM) remains the place of interest with recent years witnessing the detection of an increasing number of organic molecules, some of them being of prebiotic interest. The ISM is the matter and radiation existing between the star systems of a galaxy. It is home to clouds of gas and dust, contains primordial leftovers from the formation of the galaxy, detritus from stars, and the raw ingredients for future stars and planets.^6^

The ISM is the place where molecular evolution takes place in the galaxy. Understanding the chemical pathways active within this medium is therefore essential for tracing the origin of molecular complexity on larger astrophysical scales and for linking interstellar chemistry to the broader context of cosmic organic evolution.

It is within the cold regions of the ISM that atoms, ions, and simple molecules first assemble into more complex species, providing the raw chemical inventory later inherited by forming stars and planets.

About 85 years ago, the first evidence of molecules in space was obtained: McKellar identified sharp optical absorption lines of CN and CH using the Mount Wilson 100-in. telescope.^7^ One year later, Douglas and Herzberg found the CH cation (CH^+^) in interstellar clouds.^8^ Later, Townes argued that molecules could be detected using microwave radiation (MW).^9^ However, and perhaps surprisingly, this proposal did not receive much attention from astronomers nor – really – did the observation of CN, CH and CH^+^. Instead, astronomers held beliefs that the ISM was an environment largely devoid of molecular complexity, the discovery of simple diatomics notwithstanding. However, radioastronomy took hold in the 1960s (validating the original idea of Townes), and polyatomic molecules, both organic and inorganic in nature, were identified already at the beginning of the 1970s.

Less than 60 years ago, it became clear that the ISM is home to a diverse array of interesting polyatomic molecules. These discoveries have led to the emergence of astrochemistry, an interdisciplinary and multifaceted field at the interface of chemistry, physics, and astronomy. Astrochemistry encompasses astronomical observations and modeling, as well as theoretical and experimental laboratory investigations.^6^ Particularly fascinating is the detection of the so-called “complex organic molecules” (COMs)^10^ which has fundamentally transformed our understanding of interstellar chemistry. While regarded as an environment dominated by atoms and small diatomic species until 60 years ago, the ISM is now recognized as a site of remarkable molecular complexity.^2,10–12^ Over the past few years, observations at millimeter and submillimeter wavelengths have revealed an impressive chemical complexity,^13,14^ with the discovery of molecular species as complex as 3-hydroxy-propenal/-propanal,^15,16^ syn-glycolamide,^17^ cyanoacenaphthylene,^18^ 2-methoxyethanol,^19^ cyanopyrene,^20,21^ and cyanocoronene,^22^ just to cite some. These findings raise a central question: how does chemistry proceed so effectively under the extreme physical conditions of the ISM? Indeed, the ISM is characterized by low temperatures (ranging from 10 to 200 K), low number densities (in the 10–10^8^ cm^–3^ range), and ionizing irradiation. Under typical interstellar conditions, conventional chemical reactivity should proceed negligibly, hindered by low collision rates and insufficient thermal energy to overcome activation barriers. However, the current census of interstellar and circumstellar molecules consists of more than 340 species, with – as mentioned above – a certain degree of complexity being highlighted. The discovery that chemical reactions occur efficiently in the extreme conditions of the ISM is one of the most remarkable findings in astrochemistry.

The apparent paradox of chemical reactivity in a seemingly inert medium has driven extensive efforts aimed at unveiling interstellar chemistry. Gas-phase ion–molecule reactions were first proposed to explain molecular formation under low-temperature conditions.^23^ However, gas-phase models have proven inadequate to reproduce the full molecular complexity observed in dense clouds and star-forming regions and attention has turned to the role of the surface of interstellar grains.^11,24^ These are micrometric dust particles (mainly consisting of nonvolatile silicates) coated with icy mantles (predominantly made up of water and of other minor small species) where atoms and molecules are adsorbed. As such, interstellar grains are places where reactions can occur, also initiated by UV and/or cosmic rays,^25,26^ and can provide surface sites that facilitate nonthermal processes.^27^ Despite the progresses accomplished in the past decade, a major question remains unresolved: What governs the balance between gas-phase and surface-driven chemistry?

In this Outlook, we give an overview of the state-of-the-art in investigating interstellar chemical reactivity and highlight recent advancements. While particular emphasis will be given to the laboratory efforts that try to answer the questions raised above, we already admit that the limited space will not allow us to be as exhaustive as we would like to be. Therefore, we refer interested readers to the literature cited. Finally, in addition to current challenges, future directions will be discussed.

## Interstellar Chemistry: The Background

In the early seventies, gas-phase ion–molecule reactions were proposed to rationalize molecular abundances observed in interstellar clouds.^23^ Later, the importance of neutral–neutral reactions was recognized, even in low-temperature conditions.^28^ Subsequently, advancements in observational capabilities pointed out that astrochemical models based only on gas-phase reactivity yield abundances in strong disagreement (more than an order of magnitude) with observations.^29^ In fact, COMs began to be detected in regions where gas-phase reactions do not contribute significantly to chemical processing.^30^ Several studies have led to the recognition that chemical reactions occurring on the surface of (icy) dust grains are critical sources of molecules and that these grains, which could effectively serve as both catalyst and a sink for the deposition of chemical energy, play an important role in the chemistry of space.^26,31,32^ In particular, grain-surface chemistry is considered responsible for the efficient production of COMs.^33^ Laboratory experiments demonstrated that processing analogues of icy interstellar grains with ionizing radiation, proxies of cosmic rays and UV photons, initiates a rich chemistry.^34–36^ However, recent detections of COMs in cold environments as well as in shocked regions pointed out the fundamental role played by gas-phase chemistry.^37^

While the evidence for molecular complexity in the ISM is undisputed, there is still much to be understood about the formation of molecules and the reaction mechanisms involved. This fragmentary information also affects astrochemical modeling. Indeed, the chemical evolution of an interstellar cloud can be simulated over time using kinetic models that incorporate hundreds of reactions that involve hundreds to thousands of species.^38–40^ The need for the kinetic parameters required for the relevant reactions has led to the growth of different astrochemical databases, with KIDA^41,42^ being one of the most relevant examples. However, the data gathered in these catalogues are often estimated or extrapolated because of the fragmentary knowledge mentioned above.

The harsh conditions of the ISM pose severe constraints on the reactivity and lead to a chemistry which is often denoted as ‘exotic’ because it greatly differs from that occurring on Earth.^43,44^ The direct consequence of the low temperatures is that molecules do not have additional thermal energy (*kT*, with *k* being the Boltzmann constant and *T* the absolute temperature) and for reactions to be effective they must be exothermic. Going into the details of the reaction mechanism, the lack of thermal energy requires that reactions should proceed with a barrierless attack and submerged barriers. To fulfill these constraints, at least one of the reactants should be a highly reactive species such as a radical or an ion. In the gas phase, the very low densities of the ISM lead to the additional constraint of bimolecular products. In fact, while a unimolecular species can be produced, this is often not the final product because of the excess of energy, which cannot be removed through collisions (the very low density preventing them). Thus, since the unimolecular intermediate is unstable, it either proceeds further along the reaction path or dissociates back to reactants. Exceptions are provided by radiative stabilization, which is however usually characterized by a very low rate. Figure 1 graphically summarizes the constraints posed by interstellar conditions to chemical reactions in the gas phase. Alternatively, moving to the condensed phase, the grain can efficiently remove the excess energy, thus allowing for recombination reactions. The grain surface can also have catalytic effects and might render efficient reactions otherwise hampered by the low temperature. Finally, at the typical low temperatures of the ISM, tunneling effects should be taken into account and might open reactions otherwise blocked.

**Figure 1:**
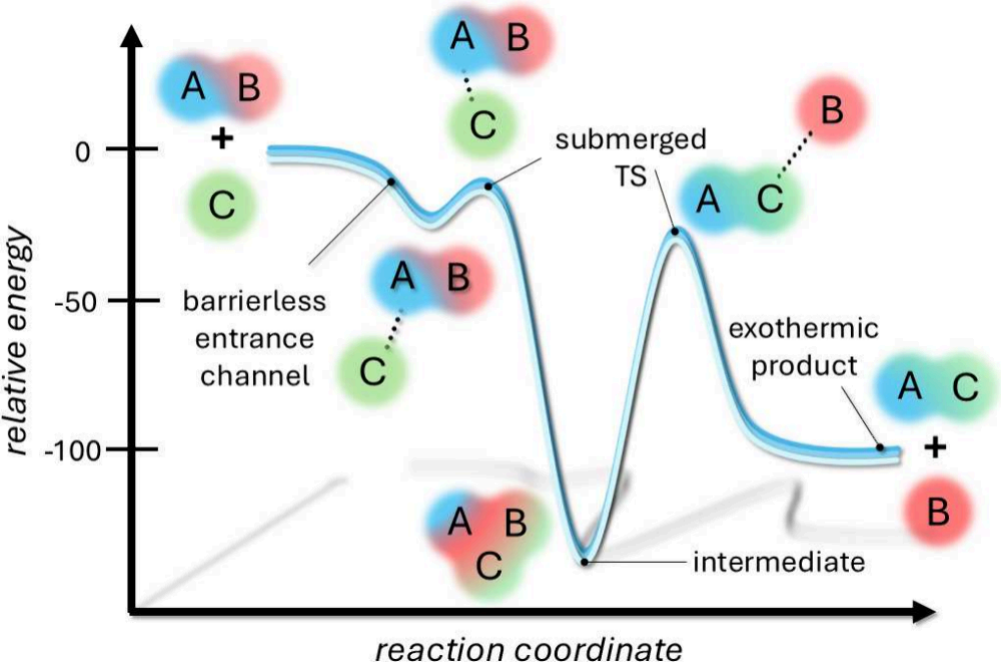
Schematic representation of the constraints posed by interstellar conditions on gas-phase reactions. Typically, the reactants, AB + C, form – without overcoming any entrance barrier– a prereactive complex (AB···C) or directly the intermediate ABC (not stabilized because of the lack of third-body effects; see text). The latter evolves, by overcoming a submerged transition state (TS, or more TSs), into the bimolecular product, AC + B.

## Unveiling Interstellar Chemistry: Current Strategies

Experiments able to reproduce the interstellar conditions are limited and, with regard to the gas phase, they are unable to reproduce at the same time the low temperature and the low pressure that are typical of the ISM. For this reason, experimental investigations of interstellar chemistry need to be supported by computational studies exploring both the thermochemical and kinetic aspects. In the following, three case studies have been selected for discussion, which starts from the case of purely computational investigations. Then, two representative sets of experimental works, one in the gas phase and one in ices, will be addressed.

## Reaction Mechanism: Exploration of Reactive Potential Energy Surfaces

While kinetic experiments remain the cornerstone for determining reaction rates, they often provide only a partial glimpse into reaction mechanisms. Not to mention the difficulties of experiments in reproducing interstellar conditions. To move beyond this limited view, a powerful complementary perspective is offered by computational chemistry.

The exploration of the reactive potential energy surface (PES) delivers the energetic landscape that connects reactants, intermediates and products, thus revealing all possible reaction channels. However, in order to be informative, computational studies should be able to exhaustively explore all possible reaction pathways with the required accuracy. The thermochemical characterization is then followed by kinetic simulations aiming at the prediction of the rate coefficients in the temperature range of interest.

Quantum chemistry provides the opportunity to investigate reactions at a molecular level with great accuracy.

The accuracy of the energetic description is critical for identifying which reaction pathways are accessible under interstellar conditions. At the extremely low temperatures characteristic of the ISM, even small uncertainties in reaction energetics or barrier heights can dramatically influence rate coefficients. The methodology developed in our research group is schematically described in Figure 2,^45–50^ which consists of five stages. The first one is the exhaustive exploration of the reactive PES. Some reactions are characterized by only a few channels containing a limited number of stationary points. An example in this respect is the gas-phase reaction between ethylene and the cyano radical (CN)

$$\begin{array}{ll} {\mathrm{CH}}_{2}={\mathrm{CH}}_{2}+\mathrm{CN} & \to {\mathrm{CH}}_{2}=\mathrm{CH}+\mathrm{HCN}\;\left(a\right)\\ & \to {\mathrm{CH}}_{2}=\mathrm{CH}+\mathrm{HNC}\;\left(b\right)\\ & \to {\mathrm{CH}}_{2}=\mathrm{CHCN}+H\;\left(c\right)\\ & \to {\mathrm{CH}}_{2}=\mathrm{CHNC}+H\;\left(d\right) \end{array}$$

which can lead, in one-step process, to hydrogen abstraction forming either HCN or HNC plus the C_2_H_3_ radical (pathways *a* and *b*, respectively) or to addition forming either vinyl cyanide or vinyl isocyanide plus H (pathways *c* and *d*, respectively) in one-/two-step process. Among these four bimolecular products, which are all exothermic, the formation route of vinyl cyanide is the only one fulfilling the two other constraints, i.e. the barrierless approach and submerged transition states (see Figure 1). In the vast majority of the cases, instead, the exhaustive exploration of a reactive network is a daunting task because of its complexity. This can easily result in unexplored reactive channels which, in turn, can affect kinetic outcomes and branching ratios. Not to mention the significant human effort that such a task might require. This has led to the introduction of computational tools for the automatic scan of reactive PESs that rely on external forces, molecular dynamics calculations, chemical heuristics or machine learning algorithms.^51–59^ However, their efficiency tends to be limited or they are unable to correctly account for constraints posed by interstellar conditions.^60,61^ Recently, we have incorporated in the methodology under discussion (Figure 2) an autonomous computational workflow (by exploiting the algorithms available in the Chemoton software^62,63^ ) capable of systematically and automatically exploring reactive PESs under interstellar conditions.^64^ This is a PES exploration tool able to account for (*i*) the exploration of all possible reaction coordinates, (*ii*) the energy limitation conditions in deciding whether to accept or not stationary points, and (*iii*) the restriction to bimolecular products. The tool was successfully applied to the oxirane (c-C_2_H_4_O) + CH reaction.^64^

**Figure 2:**
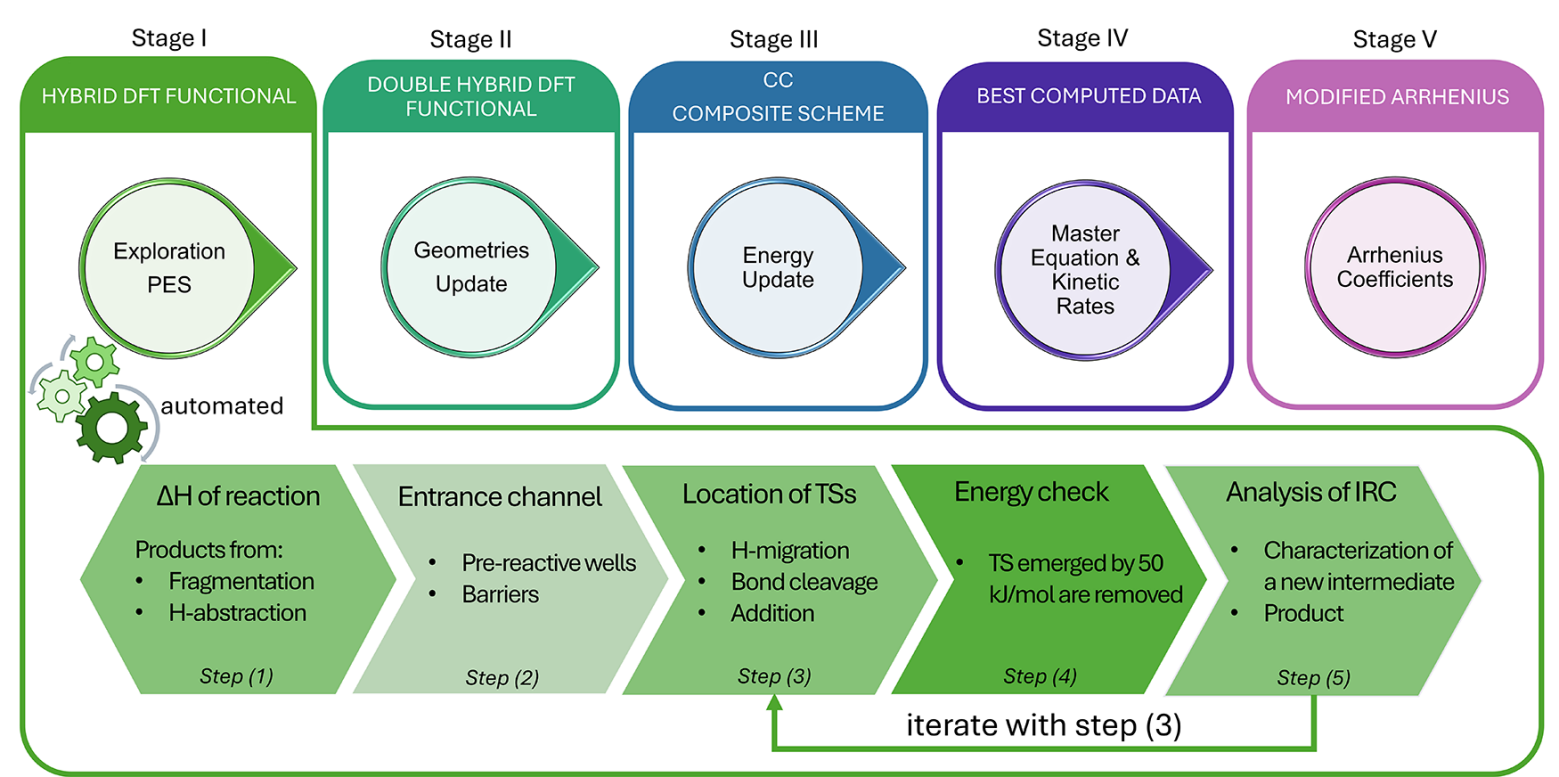
Computational scheme adopted to determine accurate and reliable kinetic rate coefficients for gas-phase reactions relevant to astrochemical modeling. The procedure consists of five sequential stages. At Stage I, the exploration of the reactive PES is carried out using a hybrid DFT functional with a small basis set, typically of double-ζ quality. As detailed in the bottom panel, this stage consists of five different steps: (1) Evaluation of the possible exothermic bimolecular products. In this step, several possible products are considered; the reaction types being considered are addition, fragmentation, H abstraction, .... For the exothermic bimolecular products, one proceeds to step (2), in which the entrance channel is explored to identify barrierless pathways and prereactive complexes. In step (3), TSs are searched from the prereactive wells, usually considering H-migration, bond cleavage, or addition processes. The relative energy (with respect to the reactants) of each TS is evaluated in step (4), and those channels having TS(s) lying more than 50 kJ·mol^–1^ above the reactants are not further considered. This threshold accounts for the uncertainty associated with the level of theory used for the PES exploration. Finally, step (5) involves the intrinsic reaction coordinate (IRC) analysis of each TS, which may lead to either a bimolecular product or a new intermediate. If the latter case applies, the procedure is iterated from step (3) until the bimolecular product is found. For each stationary point located on the reactive PES, the Hessian matrix is computed to confirm its nature (transition state or minimum). Stage I can be automated (see text). Once the PES exploration is completed, Stage II re-examines the selected portions of the reactive PES at a higher level of theory, employing a double-hybrid DFT functional in conjunction with a triple-ζ quality basis set. At this stage, the structures of the stationary points are reoptimized and harmonic zero-point energies (ZPEs) are evaluated from the updated Hessian calculations. At Stage III, electronic energies are further improved by means of composite schemes rooted in the coupled-cluster (CC) theory (the reader is referred to refs 45–50 for details). The best computed data from Stage II (geometries, vibrational frequencies, and ZPEs) and from Stage III (electronic energies) are combined in Stage IV, where the master equation (ME) is solved to obtain temperature-dependent rate coefficients. Finally, at Stage V, the computed rate constants are fitted to modified expression of the Arrhenius law in order to model their temperature dependence.

The methodology of Figure 2 suggests that, after the preliminary PES exploration, the next step is the structural improvement of the stationary points for the reaction channels that are open under interstellar conditions. This stage is followed by a further improvement of energetics, which is achieved by exploiting the best affordable level of theory (according to the dimension of the reactive system). In the last step, the kinetic simulation is performed, which leads to the rate coefficients and branching ratios.

Figure 3 gives a flavor of the methodology sketched above at work. The reaction is that between methanimine (CH_2_=NH) and the CP radical:^49^ Panel (a) shows the reactive PES and Panel (b) the kinetic simulation. The reactive PES consists of different pathways leading to six exothermic bimolecular products: PE (*E*-NHCHCP), PZ (*Z*-NHCHCP) and PN (CH_2_NCP), with H as coproduct, resulting from CP addition, and PH1 (H_2_CN), PH2 (*c*-HNCH) and PH3 (*t*-HNCH), with HCP as coproduct, resulting from H abstraction. For entirely submerged pathways a solid blue line is used, while a black trace is employed for channels with at least an emerged transition state. All addition products result from pathways open under interstellar conditions, while all abstraction products are instead ‘blocked’ by, at least, an emerged barrier. Panel (b) gives a clear idea of the impact of emerged transition states on the reaction rate. To give an example, the pathway leading to PH2 shows only one barrier emerged by ∼8 kJ·mol^–1^. This is sufficient to determine rate coefficients as small as 10^–15^ to 10^–17^ cm^3^ molecules^–1^ s^–1^. It is also evident that the height of submerged barriers can change the rate coefficients by several orders of magnitude, thus leading to a faster formation of PZ than PE.

**Figure 3:**
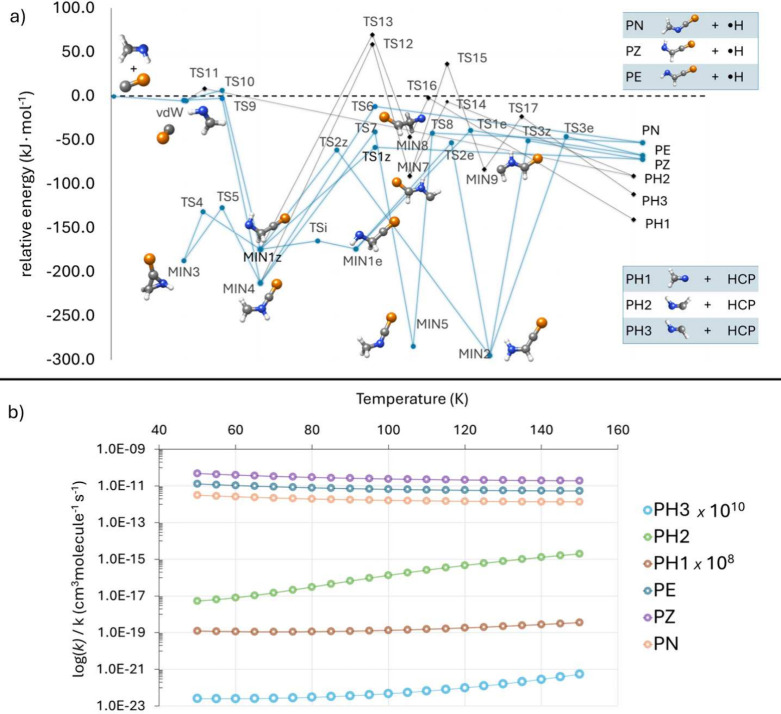
Panel (a) sketches the PES of the reaction between methanimine (CH_2_=NH) and the CP radical. The formation of the prereactive complex (vdW) is barrierless and it can only evolve into the addition products (PE, PN, and PZ) via submerged barriers (blue pathways). The formation of H-abstraction products (PH1, PH2 and PH3) involves at least one emerged TS (black pathways). Panel (b) illustrates the temperature dependence of the rate coefficients in the 50–150 K range. Production of PZ is the fastest process, followed by formation of PE and PZ. Focusing on H-abstraction, PH2 is the most favorable product, its rate coefficients being however about 6 orders of magnitude smaller than those for the formation of PN. The productions of PH1 and PH3 are even slower by several orders of magnitude.

## Formation of Benzonitrile, a Proxy for Benzene

Experimental investigations of gas-phase reactions under interstellar conditions aim to replicate as much as possible the extremely low temperatures and densities characteristic of the ISM. Experiments designed to probe gas-phase reactions at cryogenic temperatures and ultralow pressures employ techniques such as supersonic molecular beams, ion–molecule traps, and cryogenic flow cells. Coupled with ultrasensitive spectroscopic methods, including cavity ring-down spectroscopy, laser-induced fluorescence and rotational spectroscopy, these approaches allow direct observation of transient species, monitoring of reactants/products concentration, and thus deriving kinetic insights.^65–67^

In 2018, benzonitrile (c-C_6_H_5_CN) has been detected toward the TMC-1 molecular cloud.^68^ The importance of this discovery lies in the fact that benzonitrile is a good proxy of benzene, a molecule blind to radioastronomy.^2^ Benzene, in turn, is the building block of polycyclic aromatic hydrocarbons, which are widespread throughout the universe. In fact, these classes of molecules are responsible for the unidentified infrared bands.^69^ Despite their ubiquity, astronomical identification of specific aromatic molecules has proven elusive because, like benzene, they are blind to radioastronomy.^2^

The reason why benzonitrile is considered a good proxy of benzene is the efficiency of the reaction between benzene and the cyano radical, which has been investigated theoretically and experimentally.^70–73^ Some of the authors of the astronomical detection studied the reaction using rotational spectroscopy,^72^ whose intrinsic high spectral resolution and sensitivity allows for differentiating between isomers and isotopic species, even at low concentration. Although the experimental conditions in the rotational spectrometer do not replicate the very low temperature and pressure typical of the ISM, the experiment allowed the confirmation of the chemical pathways computationally derived. According to them, benzonitrile is formed from a barrierless approach of the reactants after overcoming a submerged transition state. However, its isomer, phenyl isocyanide (c-C_6_H_5_NC), is required to overcome a small entrance barrier and another emerged barrier in order to be produced. These features have been confirmed experimentally: very strong lines of benzonitrile and very weak transitions of phenyl isocyanide were observed. Later, in 2020, the reaction between CN and benzene was experimentally investigated at low temperature, in the 15–295 K range, using the CRESU technique (with CRESU being the French acronym standing for Reaction Kinetics in Uniform Supersonic Flow) combined with pulsed-laser photolysis-laser-induced fluorescence (PLP-LIF).^73^ The CRESU technique is able to reproduce very low temperatures at the price of a density which is several orders of magnitude higher than that of the ISM. The experiment demonstrated that the c-C_6_H_6_ + CN reaction is indeed rapid at temperatures relevant to the ISM and that it does not show any temperature dependence in the interval of temperature considered.

The reaction between benzene and CN was also studied, in 1999 (thus prior to benzonitrile detection), by combining crossed molecular beams (CMB) and quantum chemistry.^71^ The CMB technique allows for reproducing the one-to-one collision typical of the rarefied interstellar gas, but is conducted at room temperature. The reactive PES elaborated by Balucani et al.^71^ is similar to that of Lee et al.,^72^ the only difference lying in the entrance channel leading to the formation of phenyl isocyanide: in ref 71 a prereactive complex issuing from a barrierless approach was found with the subsequent barrier being submerged instead of being emerged as in ref 72. However, the final outcome does not change because the formation of phenyl isocyanide is hampered by the exit emerged barrier (from c-C_6_H_6_NC → c-C_6_H_5_NC + H). Computationally and experimentally, the reaction product is benzonitrile.

While the reader is referred to the cited papers for the details on the experimental techniques and the reactive PESs there obtained, the conclusion that can be drawn from those works is that the experimental investigations confirm that benzonitrile is a good proxy for benzene in the ISM and that the c-C_6_H_6_ + CN reaction needed to be included in the reaction networks modeling the chemistry of the ISM.

## Prebiotic Chemistry in Interstellar Analog Ice

In laboratory studies simulating interstellar environments, the formation of organic molecules has been shown to occur efficiently within irradiated ice analogues that mimic the icy mantles of dust grains in dense molecular clouds.^74–76^ These ices, typically composed of simple volatiles such as H_2_O, CO, CO_2_, CH_3_OH, NH_3_, and CH_4_, are subjected to energetic processing by ultraviolet photons, electrons, or ion irradiation, replicating the effects of cosmic rays. Such irradiation initiates a complex network of photochemical and radiolytic reactions, leading to the formation of reactive radicals and molecular fragments that recombine to yield more complex organic species.^74,77^ Experimental analyses, often combining infrared spectroscopy and mass spectrometry, have identified a rich suite of organic products, including alcohols, aldehydes, carboxylic acids and amino acid precursors.^26,27,34,78–82^

Grain surfaces can catalyze reactions by offering pathways with reduced energy barriers relative to the gas phase, while the bulk ice acts as an efficient energy sink, stabilizing products formed in highly exothermic processes. Together, these effects make icy mantles active participants in the molecular evolution of the interstellar medium rather than passive reservoirs of matter.^76,77^

Interstellar ices play a multifaceted role in astrochemistry: by trapping and concentrating volatile species, they bring reactants into close contact, promoting chemical reactions and possibly catalyze them.

Currently, the production of COMs is largely attributed to the chemistry occurring on interstellar grains.^83^ Since several detected COMs have a prebiotic character, tracing how these molecules form and evolve can help understand the different steps in the sequence of organizational events that could have led to the emergence of life on Earth. In this respect, laboratory studies have demonstrated the possible formation of amino acids and other prebiotic species by means of energetic processing of interstellar ices, such as UV irradiation and electron bombardment. However, the same laboratory experiments also show that these energetic processes can cause their destruction upon further irradiation.^84,85^ It is therefore of pivotal importance to understand whether prebiotic COMs can be produced by nonenergetic processes, such as radical additions or radical–radical combinations on the grain surfaces.

Focusing on glycine, the smallest amino acid, there is no proof of its presence in the ISM because of the lack of astronomical detection. However, it has been found in meteorites^86^ and comets.^87^ In 2021, Ioppolo et al.^79^ demonstrated that glycine forms in the first water-rich ice layer covering bare interstellar dust grains by means of the NH_2_CH_2_ + HOCO radical–radical recombination at 13–14 K in absence of any energetic trigger.^79^ Therefore, they showed that glycine can be produced at the early stages of star formation (prestellar), thus implying that it can be formed ubiquitously in space and be preserved in the bulk of polar ices before inclusion in meteorites and comets.^79^ Once formed, prestellar glycine can act as a precursor of more complex molecules by either energetic or nonenergetic processes.

The formation of glycine described in ref 79 assumes that the reactions occur on the surface grains. Similar surface studies have also been investigated computationally.^77^ Still focusing on glycine, Rimola and co-workers used quantum-chemical calculations to simulate the reaction occurring on water ice and leading to glycine.^88^ The icy mantle of interstellar grains was simulated using a water cluster, with the assumption of the presence of the OH radical as result of the effect of UV radiation and/or cosmic ray.^88^ The reaction of OH with CO forms the COOH radical which can further react with CH_2_=NH, widely diffuse in the ISM, to form the NHCH_2_COOH radical and then, by hydrogenation, glycine.^88^ The strategy for the computational investigation of interstellar ice chemistry is graphically presented in Figure 4. The crucial steps are (i) the reliable simulation of the water ice by means of a suitable water cluster (step 2 of Figure 4), (ii) the adsorption of the reactants on it (step 3) and (iii) the structural and energetic characterization of the reaction steps (steps 4 and 5). The point (ii) requires the identification of the binding sites and the evaluation of the strength of the ice-reactant interaction (binding energies), while the point (iii) selects the reactant pairs on the basis of their proximity and binding energy.^89^ In passing we note that, in ref 88, the presence of H_3_O^+^ and its reactions were also considered.

**Figure 4:**
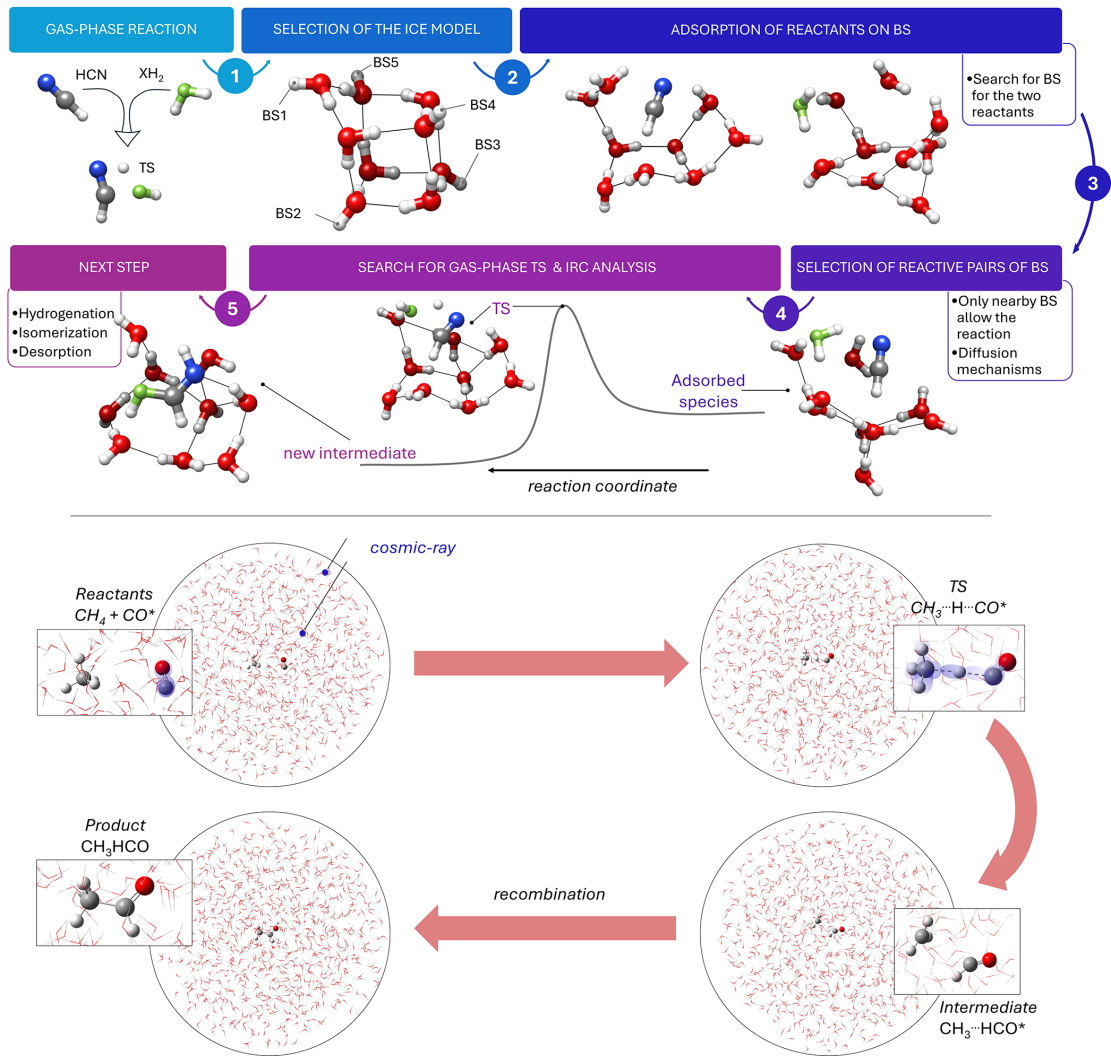
Panel (a) schematically illustrates the steps involved in the characterization of a generic reaction, HCN + XH_2_, occurring on the surface of interstellar ices. First, the gas-phase reaction is investigated to locate the corresponding TS for the association process. Then, an appropriate model is selected to describe the ice surface. Typically, the model is a cluster of water molecules or a cluster of CO species if an apolar ice is regarded. In the figure, a cluster of nine water molecules is chosen as an example. Depending on the adopted model, different binding sites (BSs) are available. The next step is the analysis of the binding energies (BEs) of the two isolated reactants adsorbed on the surface model. If two sites with large BEs are spatially close, it can be assumed that the reactants are in close proximity and react. Here, a different approach might be offered by the analysis of diffusion mechanisms of the reactants, which is however computationally more expensive. Using a cluster with the two reactants adsorbed as starting point, the TS is searched using that of the gas-phase reaction as a guidance. Then, the IRC analysis is employed to find the next intermediate of the reaction, which stabilizes if it lies lower in energy than the reactants. The intermediate might undergo further chemical processing, like hydrogenation or isomerization, or simply desorbes if the available energy exceeds its BE. Panel (b) shows a possible scheme for the reaction between CH_4_ and CO occurring inside the icy mantle of an interstellar grain. In this case, the cosmic rays penetrate the bulk and provide the energy for the reaction to take place in a concerted manner (with at least one species being activated by irradiation), thus leading to the final product (CH_3_CHO).

Recent works suggest that interstellar grain chemistry may occur within the bulk of icy mantles rather than solely on their surfaces. Laboratory studies by Ralf I. Kaiser and co-workers have shown that energetic processing through cosmic-ray bombardment can penetrate deep into astrophysical ice analogues, driving bond cleavage and radical formation throughout the ice matrix.^34^ These findings challenge the traditional view of surface-limited reactivity, revealing that the interior of interstellar ices can host rich, radiation-driven chemistry that contributes directly to the synthesis of COMs in space. The investigation of such ice-interior processes in the laboratory and the subsequent inclusion of the obtained data (rate constants, reaction products, branching ratios, ...) into astrochemical reaction networks has allowed astrochemical models to better match the astronomical abundances, thus suggesting that formation routes of COMs on interstellar grains were previously overlooked.^78,90,91^ Therefore, laboratory experiments exploiting experimental techniques able to probe the formation of COMs in interstellar ice analogues via interaction of ionizing radiation are crucial to unravel comprehensively the complex organic chemistry occurring in the ISM. To give some examples, Kaiser and co-workers demonstrated the production, in interstellar ice analogs, of lactic acid – a key biorelevant hydroxycarboxylic acid which is ubiquitous in living organisms,^92^ glycinal (HCOCH_2_NH_2_) and acetamide – simple molecular building blocks of biomolecules in prebiotic chemistry,^80^ glyceric acid – the simplest sugar acid which is a key molecule in biochemical processes,^81^ and carbamic acid – a source of the molecular building blocks for more complex proteinogenic amino acids.^93^

While computational studies of surface reactions are well-established and widely employed, also in support of the corresponding experiments,^77^ the methodology required to investigate bulk-ice reactions is still not well-defined.

In passing we note that reactivity on the bare grains has also been considered, thus further exploiting their potential as chemical catalysts.^94^ Interstellar grains appear in every phase of star and planet formation. Over time, they acquire icy coatings in the dense, cold regions of space. However, recent studies suggest that, because their surfaces are porous and uneven, parts of the dust core remain exposed to the gas around them,^95^ allowing a complex interplay between bare silicates and gas phases.

## Future Directions and Conclusions

A flavor of the remarkable advances achieved in uncovering interstellar chemistry and of the methodologies that enabled them has been provided in the previous section. Despite the recent progresses, the study of interstellar chemistry remains in its infancy.^96–99^ Astronomical observations have revealed a surprising molecular richness in even the coldest and most diffuse regions of space, yet only a fraction of the detected species can be fully explained by current chemical models.^100^

The underlying reaction networks are often poorly constrained, and laboratory measurements under true interstellar conditions are scarce. At present, our understanding of interstellar chemistry is more descriptive than predictive.^43,99,101–103^ Observations continue to reveal molecules whose origins challenge existing paradigms and expose the limitations of standard reaction networks. Although powerful new tools are beginning to bridge this gap, a comprehensive, quantitative picture of chemical evolution in space remains elusive.

The interplay between gas-phase reactions and grain-surface processes adds further complexity, demanding experimental and theoretical frameworks that can span vast differences in time, temperature, and density. As said, the previous section addressed some exemplificative studies in view of presenting some current strategies to study the interstellar chemistry. Production of molecules in the ISM however arises from a delicate interplay between reactions in the gas phase and those occurring on the surfaces/in the bulk of dust grains.^104^ Understanding this coupling is therefore crucial and might be mandatory to explain the abundance of some detected molecular species. In this respect, experiment or computational studies that simulate the coexistence of the two types of reactivity, gas and grain, are needed.

The integration of experiment and theory will be the key to future progress in interstellar chemistry.^82,99,100,105^ On the experimental side, advances in cryogenic laboratory astrophysics are expected to refine our understanding of surface reactions on dust analogues under true interstellar conditions. On the theoretical front, increasingly sophisticated multiscale models will be key to bridging the gap between atomistic mechanisms and astrophysical observables. High-level quantum-chemical methodologies combined with machine-learning approaches will open the way to accurately uncover an increasing number of reactive processes in systems of increasing complexity, while kinetic simulations will continue to connect microscopic dynamics to macroscopic chemical evolution.

Ultimately, the interplay of experiment, computation, and observation will yield a predictive astrochemistry, able to explain the molecular richness of the interstellar medium.

In this respect, The James Webb Space Telescope (JWST) will play a pivotal role in unveiling the chemistry of interstellar ices. Its observations are providing the first comprehensive view of ice composition, structure, and evolution under astrophysical conditions.^106–108^ Therefore, it will offer the possibility to link solid-phase chemistry on dust grains and the gas-phase molecules released during star formation processes. On the other hand, the SKA-Mid component (0.35–15.4 GHz) of the Square Kilometer Array observatory (SKA-Mid in South Africa and SKA-Low in Australia) will provide a step change in the search for gas-phase prebiotic molecules in the interstellar medium by combining exceptional sensitivity with access to centimeter-wavelength rotational transitions that are inaccessible or confusion-limited in the millimeter-wave region. This capability will enable the detection of larger and more complex organic species, including key precursors to biologically relevant molecules.^109^ Therefore, SKA-mid is expected to extend the ALMA (Atacama Large Millimeter/submillimeter Array) observations at millimeter/submillimeter wavelengths that have revealed a rich inventory of complex organic and prebiotic molecules in Sun-like protostars, protoplanetary disks, and star-forming regions.

Interstellar chemistry is currently going through a significant transformation: decades of observational, experimental, and theoretical progress have revealed a molecularly rich and dynamically evolving universe, but the mechanisms driving this complexity are only partially understood. The synergy between astronomical observations, laboratory experiments and increasingly sophisticated computational models promises to bridge these gaps. These efforts are shifting astrochemistry into a predictive framework capable of tracing the chemical evolution from interstellar clouds to planetary systems.

## Supplementary Material



## Notes

Transparent Peer Review report available (PDF)
