# Supplementary material for: Chemistry in Extreme Environments: The Mystery of Molecular Complexity in Space
Source: ACS Cent Sci. 2026 Jan 30;12(2):174–84. doi: 10.1021/acscentsci.5c02122 (PMC12956029; doi:10.1021/acscentsci.5c02122)
Supplement: Supplementary file 1 [file oc5c02122_si_001.pdf]

oc-2025-02122t.R1

Name: Peer Review Information for "Chemistry in Extreme Environments: The Mystery of Molecular Complexity in Space"

First Round of Reviewer Comments

Reviewer: 1

Comments to the Author

Review of oc-2025-02122t by Puzzarini and Silvia: “Chemistry in Extreme Environments: The Mystery of Molecular Complexity in Space”

This Outlook-style paper is a nice overview of the current status and progress in the field of astrochemistry, with specific emphasis on the authors’ area of expertise in reaction modeling through transition state theory. It is well written with clear diagrams that add value to the paper for the reader, and overall this work provides a good review of the topic.

I do not have any criticisms that would prevent publication, however I noted in some places where the English could be improved. These modifications can take place by the authors or the journal copyeditors.

Otherwise, I recommend the paper for publication in ACS.

Conor Nixon

Reviewer: 2

Comments to the Author

The manuscript presents an insightful and well-written Outlook on interstellar chemistry and the formation of molecular complexity in space. The work is timely, clearly structured, and certainly worthy of publication in ACS Central Science. Before acceptance, however, I would like the authors to address the following comments:

Page 4 (end of right column):

The authors state that the computational tools cited in refs. 51–56 “are based on ML algorithms.” However, these methods predominantly rely on external forces, MD simulations, or chemical heuristics rather than machine learning. The authors should correct or refine this statement.

Page 7 (left column, top):

The authors mention that the methods in refs. 57 and 58 are inefficient or inadequate for accounting for ISM constraints and seem to imply that Chemoton overcomes these limitations. If this is the case, the authors should explicitly state the advantages of Chemoton over those approaches. Otherwise, the paragraph should be rewritten to clarify that Chemoton was simply selected as the method of choice, without implying clear methodological superiority.

Grammatical and typographical corrections

Figure 1 legend:

“…by overcoming a submerged transition state…”

Page 7 (left column, bottom):

“It is also evident that the height of submerged barriers can change the rate coefficients by several orders of magnitude…”

Page 7 (right column, top):

“The importance of this discovery lies in the fact that benzonitrile...”

Page 8 (left column, bottom):

“...the conclusion that can be drawn from those works is that...”

Once these minor points have been satisfactorily addressed, I fully endorse this Outlook and recommend its publication in ACS Central Science.

Reviewer: 3

Comments to the Author

The article of Puzzarini and Alessandrini is an understanding work on the State-of-the-art of computational astrochemistry. The work is clear, well-structured and highly understandable. It can be used as useful work for researchers who aim to be introduced in this field.

I have no reservations to recommend this work to be published in ACS Central Science. However, I encourage the authors to take the following comment into account with the aim of improving its quality.

- Page 2 of 18, line 9, right column: coated with icy mantles of water -> coated with icy mantles predominantly made up of water (but also of other minor species like CO, CO2, NH3 and CH3OH).

- Page 2 of 18, line 12, right column: “catalytic sites”. I disagree with this term because, as already mentioned later in the text by the same authors, grains present different roles beyond actual chemical catalytic sites. Thus, I would replace “can act as catalytic

- sites” by “can present surface reaction sites that can help reaction progress”. Later, this idea is well-developed.

- Age 5 of 18, lines 37-42, right column: I think that rather than describing the reactions, it would be better to write the reaction equations.

- A format aspect of Figure 2: I think it would be clearer if the outlines of the Stages are thicker. This will allow differentiating better the components of Stage I (which includes the bottom panel) from the rest of the Stages.

- Page 8 of 18, line 44, left column: those work -> those Works

- Page 8 of 18, line 41, right column: I find the sentence od “understand how life originated on Earth” too strong. I suggest softening it by “can help understand different steps in the sequence of organizational events that could have led to the emergence of life on Earth.”

- In the conclusions section authors talk about the relevance of JWST for ices. But I missed similar comments for ALMA and the forthcoming SKAO for gas phase molecules. I think the work would benefit if authors mention anything on these facilities.

Reviewer: 4

Comments to the Author

This 'outlook' review provides a brief but useful perspective of the status of astrochemistry research. It summarises the field and has an extensive reference list. The article is not controversial its conclusions being widely accepted by the community. However I would comment on one aspect. The authors state that there is a recognition that chemistry on ISM ice covered dust grains is not just a surface process (true) but a bulk effect and use R Kaiser experiments as revealing this. This is not quite accurate Kaiser is using electrons as cosmic ray mimic but cosmic rays are mainly ions and it is well known that ions deposit their energy at depth through their stopping power and the Bragg peak. Ion induce chemistry in ISM ices is well established and present work at Ganil facilty in Caen France and Atomki Hungary and Catania, Italy (amongst others) has demonstrated just such bulk chemistry in detail, This work and the role of ions should be included and discussed in the article,

Author's Response to Peer Review Comments:

REPLY TO REVIEWERS’ COMMENTS

Reviewer(s)' Comments to Author:

Reviewer: 1

Recommendation: Publish in ACS Central Science after minor revisions noted.

Comments:

Review of oc-2025-02122t by Puzzarini and Silvia: “Chemistry in Extreme Environments: The

Mystery of Molecular Complexity in Space”

This Outlook-style paper is a nice overview of the current status and progress in the field of astrochemistry, with specific emphasis on the authors’ area of expertise in reaction modeling through transition state theory. It is well written with clear diagrams that add value to the paper for the reader, and overall this work provides a good review of the topic.

R: We thank the reviewer for the very positive feedback.

I do not have any criticisms that would prevent publication, however I noted in some places where the English could be improved. These modifications can take place by the authors or the journal copyeditors.

R: We have incorporated all the modifications suggested by the reviewer.

Otherwise, I recommend the paper for publication in ACS.

Conor Nixon

Additional Questions:

Quality of experimental data, technical rigor: Top 10%

Significance to chemistry researchers in this and related fields: Top 1%

Broad interest to other researchers: Top 1%

Novelty: Top 10%

Is this research study suitable for media coverage or a First Reactions (a News & Views piece in the journal)?: No

Reviewer: 2

Recommendation: Publish in ACS Central Science after minor revisions noted.

Comments:

The manuscript presents an insightful and well-written Outlook on interstellar chemistry and the formation of molecular complexity in space. The work is timely, clearly structured, and certainly worthy of publication in ACS Central Science.

R: We thank the reviewer for the very positive feedback.

Before acceptance, however, I would like the authors to address the following comments:

Page 4 (end of right column):

The authors state that the computational tools cited in refs. 51–56 "are based on ML algorithms." However, these methods predominantly rely on external forces, MD simulations, or chemical heuristics rather than machine learning. The authors should correct or refine this statement.

R: We agree with the reviewer, and we thank him/her for the comment. The methods have been specified and we added further references specifically on machine learning. The sentence now reads:

"This has led to the introduction of computational tools for the automatic scan of reactive PESs that rely on external forces, molecular dynamics calculations, chemical heuristics or machine learning algorithms.[51-59]" The added references are:

Kayala, M. A.; Baldi, P. ReactionPredictor: Prediction of Complex Chemical Reactions at the Mechanistic Level Using Machine Learning. J. Chem. Inf. Model. 2012, 52, 2526–2540.

Gao, C. W.; Allen, J. W.; Green, W. H.; West, R. H. Reaction Mechanism Generator: Automatic Construction of Chemical Kinetic Mechanisms. Comput. Phys. Commun. 2016, 203, 212–225.

Liu, Y.; Morrow, J. D.; Ertural, C.; Fragapane, N. L.; Gardner, J. L. A.; Naik, A. A.; Zhou, Y.; George, J.; Deringer, V. L. An automated framework for exploring and learning potentialenergy surfaces. Nat. Commun. 2025, 16, 7666.

Page 7 (left column, top):

The authors mention that the methods in refs. 57 and 58 are inefficient or inadequate for accounting for ISM constraints and seem to imply that Chemoton overcomes these limitations. If this is the case, the authors should explicitly state the advantages of Chemoton over those approaches. Otherwise, the paragraph should be rewritten to clarify that Chemoton was simply selected as the method of choice, without implying clear methodological superiority.

R: We agree with the reviewer, and we thank him/her for the comment. We have made the details of our implementation that exploits Chemoton. The text now reads:

"Recently, we have incorporated in the methodology under discussion (Figure 2) an autonomous computational workflow (by exploiting the algorithms available in the Chemoton software[62,63]) capable of systematically and automatically exploring reactive PESs under interstellar conditions.[64] This is a PES exploration tool able to account for (i) the exploration of all possible reaction coordinates, (ii) the energy limitation conditions in deciding whether to accept or not stationary points, and (iii) the restriction to bimolecular products. The tool was successfully applied to the oxirane (c-C_2H_4O) + CH reaction.[64]"

Note. Reference 64 has been updated because it has been accepted for publication and it is now at a production stage with DOI assigned.

Grammatical and typographical corrections

Figure 1 legend:

"…by overcoming a submerged transition state…"

R: This correction has been incorporated.

Page 7 (left column, bottom):

“It is also evident that the height of submerged barriers can change the rate coefficients by several orders of magnitude...”

R: This correction has been incorporated.

Page 7 (right column, top):

“The importance of this discovery lies in the fact that benzonitrile...”

R: This correction has been incorporated.

Page 8 (left column, bottom):

“...the conclusion that can be drawn from those works is that...”

R: This correction has been incorporated.

Once these minor points have been satisfactorily addressed, I fully endorse this Outlook and recommend its publication in ACS Central Science.

Additional Questions:

Quality of experimental data, technical rigor: Top 1%

Significance to chemistry researchers in this and related fields: Top 1%

Broad interest to other researchers: Top 10%

Novelty: Top 10%

Is this research study suitable for media coverage or a First Reactions (a News & Views piece in the journal)?: No

Reviewer: 3

Recommendation: Publish in ACS Central Science after minor revisions noted.

Comments:

The article of Puzzarini and Alessandrini is an understanding work on the State-of-the-art of computational astrochemistry. The work is clear, well-structured and highly understandable.

It can be used as useful work for researchers who aim to be introduced in this field.

I have no reservations to recommend this work to be published in ACS Central Science.

R: We thank the reviewer for the very positive feedback.

However, I encourage the authors to take the following comment into account with the aim of improving its quality.

- Page 2 of 18, line 9, right column: coated with icy mantles of water -> coated with icy mantles predominantly made up of water (but also of other minor species like CO, CO2, NH3 and CH3OH).

R: This correction has been incorporated. However, we have made the sentence shorter. This now reads:

"… coated with icy mantles (predominantly made up of water and of other minor small species)."

We have not specified the examples of minor species because ice composition is detailed later in the text.

- Page 2 of 18, line 12, right column: "catalytic sites". I disagree with this term because, as already mentioned later in the text by the same authors, grains present different roles beyond actual chemical catalytic sites. Thus, I would replace "can act as catalytic sites" by "can present surface reaction sites that can help reaction progress". Later, this idea is welldeveloped.

R: This correction has been incorporated. However, since – as mentioned by the reviewer – the different roles played by the ice surfaces are detailed later in the text, we have kept the sentence shorter. This now reads as:

"… and can provide surface sites that facilitate non-thermal processes."

- Page 5 of 18, lines 37-42, right column: I think that rather than describing the reactions, it would be better to write the reaction equations.

R: This correction has been incorporated. As suggested by the reviewer, the reaction equations have been introduced in the text, which now reads:

"An example in this respect is the gas-phase reaction between ethylene and the cyano radical (CN)

$$CH_2{=}CH_2 + CN \rightarrow CH_2{=}CH + HCN \text{ (a)}$$

$$\rightarrow CH_2{=}CH + HNC \text{ (b)}$$

$$\rightarrow CH_2{=}CHCN + H \text{ (c)}$$

$$\rightarrow CH_2{=}CHNC + H \text{ (d)}$$

which can lead, in one-step process, to hydrogen abstraction forming either HCN or HNC plus the C2H3 radical (pathways a and b, respectively) or to addition forming either vinyl cyanide or vinyl isocyanide plus H (pathways c and d, respectively) in one-/two-step process. Among these four bimolecular products, which are all exothermic, the formation route of vinyl cyanide is the only one fulfilling the two other constraints, i.e. the barrierless approach and submerged transition states (see Figure 1)."

- A format aspect of Figure 2: I think it would be clearer if the outlines of the Stages are thicker. This will allow differentiating better the components of Stage I (which includes the bottom panel) from the rest of the Stages.

R: This correction has been incorporated. Indeed, the figure is now clearer.

- Page 8 of 18, line 44, left column: those work -> those Works R: This correction has been incorporated.

- Page 8 of 18, line 41, right column: I find the sentence od "understand how life originated on Earth" too strong. I suggest softening it by "can help understand different steps in the sequence of organizational events that could have led to the emergence of life on Earth." R: This correction has been incorporated.

- In the conclusions section authors talk about the relevance of JWST for ices. But I missed similar comments for ALMA and the forthcoming SKAO for gas phase molecules. I think the work would benefit if authors mention anything on these facilities.

R: We thank the reviewer for this comment. The added text (soon after that addressing the relevance of JWST) is:

“On the other hand, the SKA-Mid component (0.35-15.4 GHz) of the Square Kilometer Array observatory (SKA-Mid in South Africa and SKA-Low in Australia) will provide a step change in the search for gas-phase prebiotic molecules in the interstellar medium by combining exceptional sensitivity with access to centimeter-wavelength rotational transitions that are inaccessible or confusion-limited in the millimeter-wave region. This capability will enable the detection of larger and more complex organic species, including key precursors to biologically relevant molecules.[109] Therefore, SKA-mid is expected to extend the ALMA (Atacama Large Millimeter/submillimeter Array) observations at millimeter/submillimeter wavelengths that have revealed a rich inventory of complex organic and prebiotic molecules in Sun-like protostars, protoplanetary disks, and star-forming regions.”

Additional Questions:

Quality of experimental data, technical rigor: Top 10%

Significance to chemistry researchers in this and related fields: Top 10%

Broad interest to other researchers: Top 10%

Novelty: Top 10%

Is this research study suitable for media coverage or a First Reactions (a News & Views piece in the journal)?: No

Formatting Needs:

Pull Quotes (Outlook): We encourage you to select 3 - 4 quotes from your Outlook that you would like highlighted in your paper. The quotes should be one sentence-long, unique to the Outlook and not from previously cited work. Please list your quotes at the end of the manuscript file.

R: The pull quotes have been reduced to four. They are highlighted along the text by a lightgrey box and boldface font as well as listed at the end of the manuscript (after the reference list, before the TOC graphics).
